# BEHAVIORAL DESIGN: A NOVEL APPROACH TO CREATING ADVANCE DIRECTIVE INTERVENTIONS

**DOI:** 10.1093/geroni/igz038.479

**Published:** 2019-11-08

**Authors:** Elise Tarbi, Brianna Morgan, Jason Sloan

**Affiliations:** 1 University of Pennsylvania School of Nursing, Philadelphia, Pennsylvania, United States; 2 University of Pennsylvania Law School, Philadelphia, Pennsylvania, United States

## Abstract

The 1.7 million older adults receiving long-term services often do not receive end of life care consistent with their wishes. Advance directives (ADs) can help, yet only one-third of Americans have completed ADs. The limited effectiveness of traditional interventions to increase AD completion may be because they do not address the behavioral aspects of advance care planning. Behavioral Design is an innovative approach that combines design thinking and behavioral economics to identify predictable behavioral bottlenecks and create real-time solutions. This study used Behavioral Design to address low AD completion rates of long-term care residents. Consistent with the Behavioral Design process, an interdisciplinary team compiled evidence from 10 diverse data sources to identify behavioral bottlenecks to AD completion. These barriers were analyzed using the cognitive bias codex to determine behavioral levers for intervention. Informed by these findings, the study team designed multicomponent interventions to address behavioral aspects of AD completion. Four behavioral bottlenecks incorporating ten behaviorally mediated causes for lack of AD completion were identified. For example, AD completion is affected by complexity mediated by hassle factor, choice overload, and ambiguity effect. Three interventions were designed to address these behaviorally mediated causes. For example, the intervention HeAD Start would provide a simple, easy to read AD (addressing choice overload) to residents upon admission (addressing hassle factor) with scheduled follow-up by trained staff (addressing ambiguity effect). Behavioral Design incorporates design thinking and leverages behavioral economic principles to create behaviorally mediated AD interventions. Next steps include testing behaviorally informed designs in practice.

